# Mesenchymal stem/stromal cell function in modulating cell death

**DOI:** 10.1186/s13287-019-1158-4

**Published:** 2019-02-13

**Authors:** Abderrahim Naji, Benoit Favier, Frédéric Deschaseaux, Nathalie Rouas-Freiss, Masamitsu Eitoku, Narufumi Suganuma

**Affiliations:** 1 Department of Environmental Medicine, Cooperative Medicine Unit, Research and Education Faculty, Medicine Science Cluster, Kochi Medical School (KMS), Kochi University, Kohasu, Oko-Cho, Nankoku City, Kochi Prefecture 783-8505 Japan; 2 CEA-Université Paris Sud INSERM U1184, IDMIT Department, IBFJ, DRF, Fontenay-aux-Roses, France; 3 STROMALab, UMR 5273 CNRS, INSERM U1031, Etablissement Français du Sang (EFS) Occitanie, Université de Toulouse, Toulouse, France; 4 CEA, DRF-Institut Francois Jacob, Division de recherche en hématologie et immunologie (SRHI), Hôpital Saint-Louis, Paris, France

**Keywords:** Mesenchymal stem cell, Cell function, Cell death, Cell therapy

## Abstract

Mesenchymal stem/stromal cells (MSCs) delivered as cell therapy to individuals with degenerative and/or inflammatory disorders can help improve organ features and resolve inflammation, as demonstrated in preclinical studies and to some extent in clinical studies. MSCs have trophic, homing/migration, and immunosuppression functions, with many benefits in therapeutics. MSC functions are thought to depend on the paracrine action of soluble factors and/or the expression of membrane-bound molecules, mostly belonging to the molecular class of adhesion molecules, chemokines, enzymes, growth factors, and interleukins. Cutting-edge studies underline bioactive exchanges, including that of ions, nucleic acids, proteins, and organelles transferred from MSCs to stressed cells, thereby improving the cells’ survival and function. From this aspect, MSC death modulation function appears as a decisive biological function that could carry a significant part of the therapeutic effects of MSCs. Identifying the function and modes of actions of MSCs in modulating cell death may be exploited to enhance consistency and efficiency of cell therapy that is based on MSCs as medical treatment for degenerative and/or inflammatory diseases. Here, we review the essentials of MSC functions in modulating cell death in unfit cells, and its modes of actions based on current advances and outline the clinical implications.

## Background

Mesenchymal stem/stromal cells (MSCs) are isolated from different biological sources and expanded ex vivo in culture. These MSC cultures are thought to contain diverse cell subsets resulting from intrinsic and extrinsic influences in addition to inherent disparities related to sources and donors [1–5]. The MSC identity is under scrutiny [6], despite a consensus for the minimum criteria to identify MSCs proposed a decade ago by the International Committee for Cell Therapy (ISCT) [7]: (1) MSCs must be adherent and proliferate in vitro under standard culture conditions; (2) MSCs must feature surface expression of cluster of differentiation (CD)105, 73, and 90 but not CD45, 34, 14, 11b, 79α, and 19, or human leucocyte antigen-DR; and (3) MSCs must, upon suitable stimulation in vitro, demonstrate an ability to differentiate into adipocytes, chondroblasts, and osteoblasts. Since then, the ISCT criteria have been used to assess the MSC identity in preclinical and clinical studies but often because of lack of alternative methods for identifying MSCs per se with explicit biomarkers [6, 8–10]. However, both scientists and clinicians alike acknowledge that cell heterogeneity is to be expected in any ex vivo MSC cultures used in preclinical and clinical settings [2, 4, 5, 11–14]. MSCs from different biological sources (i.e., from the bone marrow [BM-MSCs], adipose tissue [ASCs], or umbilical cord [UC-MSCs]), a fortiori are not alike, but these MSCs in ex vivo cultures might share common features in agreement with the ISCT criteria [5, 15].

The identification of unambiguous biomarkers to select identical MSCs regardless of source, donor, or any other variables is critical to develop MSC therapy [6]. Therefore, investigations of MSC identity remain crucial in the search for specific biomarkers to define MSC identity in vivo and ex vivo. Several works have attempted to sort MSCs with the use of “stemness” biomarkers by targeting surface antigens such as STRO-1, stage-specific embryonic antigen 1 (SSEA-1), SSEA-4, CD271, or CD146 [6]. Still, no marker has shown a unique specificity for identifying MSCs per se [6, 16].

Despite these hurdles in coining MSC identity, knowledge of MSC functions is advancing rapidly, conveying other means to assess MSCs in vitro according to their actual biological functions, which can also predict the therapeutic potency of MSCs in vivo [8, 9, 17, 18]. Usually, ex vivo-expanded MSCs are considered to exhibit five biological functions of interest in therapy [7, 19–27]: (1) proliferation, (2) multipotency, (3) homing/migration, (4) trophic ability, and (5) immunosuppression, often examined independent of each other. Scientific advances have provided further understanding of modes of actions of each MSC function [1, 19, 25, 27–30]. Yet, MSC functions remain incompletely defined because of the complexity and diversity in regulation and/or modes of actions of each MSC function considered individually as well as overlaps in biological effects [17, 27, 31, 32].

Here, we discuss a sixth function of MSCs—death modulation. We focus predominately on the death modulation function of MSCs obtained from different species and biological sources, its modes of actions, and its clinical implications for human MSCs to be exploited for degenerative and/or inflammatory diseases [33, 34].

### Regulated cell death in diseases

Regulated cell death (RCD) is a fundamental biological process controlling cell fate in health and diseases [33–35]. RCD largely consists of apoptosis, necroptosis, and pyroptosis, among the most deciphered cell death modes [36]. Apoptosis represents an RCD whose execution depends on caspases-3/6/7, whereas mixed lineage kinase domain-like and gasdermin D proteins execute necroptosis and pyroptosis, respectively [36]. Uncontrolled RCD in diseases amplifies tissue damage and inflammation, which in turn could result in permanently impaired organ functions [33]. Hence, RCD is often engaged in undesirable events prolonging degenerative and/or inflammatory diseases [33]. However, cells that are resistant to RCD might participate in tumor growth [37]. In both cases, RCD pathways represent relevant therapeutic targets for numerous disorders.

### Biological function of MSCs in modulating cell death

Recently, an increasing number of studies have emphasized the ability of MSCs to promote cell rescue or cell survival of injured adult stem cells or somatic cells, enduring early signaling of RCD, via multifaceted modes of actions both in vitro and in vivo [38–60]. MSC functioning to modulate processes of RCD could directly aid in restraining damage caused to organs in diseases and/or indirectly restraining the release of harmful factors by dying cells, thereby avoiding the amplification of deleterious inflammation, additional tissue damage, and loss of organ function [33]. The death modulation function of MSCs should be considered separately from their trophic function or immunosuppression function, because the modes of actions may be independent, although the biological effects might be intertwined [17].

Actually, evidence shows that MSCs modulate RCD occurring in third-party cells, especially those affected by apoptosis, necroptosis, and pyroptosis [17, 39, 41, 46–48, 50, 53, 54, 56, 57, 61, 62]. The MSC death modulation function has been rarely identified as such, and conceivably, this function is confused with the MSC trophic function or immunosuppression function because of overlap in biological effects that can be individually attributed to each of these functions [17]. Accordingly, several preclinical studies indicated that MSCs are susceptible to cellular interactions with adult stem cells, progenitors, or somatic cells, such as hematopoietic stem cells (HSCs) or cardiomyoblasts, alveolar epithelial cells, cardiomyocytes, endothelial cells, macrophages, and neurons, favoring cell survival [39, 41, 46–48, 50, 53, 54, 56, 57, 61, 62]. Here, we discuss the modes of actions that mediate the MSC death modulation function.

### Modes of actions of MSC death modulation function

The MSC death modulation function can be envisaged via different elaborated modes of actions that could involve (1) secreted paracrine factors [39, 42, 45, 47, 48, 50, 54–58, 61, 63–65] (Table 1), most likely (2) Ca^2+^ ion exchange via the connexin (Cx)-43 gap junction [41, 43, 60, 66–68] (Table 2), (3) transfer of mitochondria via tunneling nanotubes (TNTs) [27, 38, 41, 43, 44, 46, 69] (Table 3), and (4) transfer of bioactive microRNAs (miRNAs) and/or proteins via extracellular vesicles (EVs) [27, 40, 43, 46, 51, 53, 62, 70–73] (Table 4) from MSCs to RCD-affected cells. However, it should be noted that modes of actions of MSCs in modulating cell death of unfit cells that encompass secretion of paracrine factors and/or other pathways remain uncertain (Table 1). By contrast, modes of actions depending on gap junctions, TNTs, and EVs appear dedicated specifically to rescue cells from death pathways. Thereby, we evoke paracrine factors and/or other pathways in MSC death modulation function, but we focus explicitly on modes of actions implying intercellular communications such as gap junctions, TNTs, and EVs between MSCs and unfit cells.

**Table 1:** Mesenchymal stem/stromal cell (MSC) death modulation function depending on paracrine factors and/or other modes of actions

Studies	Sources of MSCs	MSC death modulation function	Modes of actions	References
Preclinical in vitro and in vivo	Human BM-MSCs	Apoptosis in human primary CD34+ cells induced by γ-irradiation with xenotransplantation in a baboon model	Not determined	Drouet et al. [42]
Preclinical in vitro	Human BM-MSCs	Activation-induced cell death, apoptosis induced by anti-CD3 Abs, or deprivation of serum or anti-Fas Abs in primary human thymocytes and human Jurkat T cell line	Fas-FasL pathway inhibition	Benvenuto et al. [39]
Preclinical in vitro	Human BM-MSCs	Apoptosis in human primary neutrophils induced by IL-8, or in resting state	IL-6, STAT3	Raffaghello et al. [58]
Preclinical in vitro	Rat BM-MSCs	Apoptosis in Rat PC12 neuron cell line and rat primary cortical neurons induced by deprivation of serum and exposure to EtOH	PI3K/Akt, ERK1/2	Liu et al. [45]
Preclinical in vitro	Mouse and human BM-MSCs	Apoptosis and/or necroptosis induced in rat PC12 neuron cell line, human ReNcell CX neural progenitor cell line, and rat cortical primary neurons induced by 6-OHDA	Prosaposin	Li et al. [65]
Preclinical in vitro	Human UC-MSCs	Apoptosis in human primary neutrophils induced by deprivation of serum	Not determined	Maqbool et al. [47]
Preclinical in vitro	Rat BM-MSCs	Apoptosis in human SH-SY5Y neuroblastoma cell line induced by misfolded tau protein	Not determined	Zilka et al. [56]
Preclinical in vitro	Mouse and human BM-MSCs	Apoptosis in rat primary cortical neurons induced by deprivation of glucose and oxygen	PI3K/Akt, STAT3	Scheibe et al. [50]
Preclinical in vivo	Rat BMMSCs	Apoptosis in rat lung fibroblasts induced by cigarette smoke extract	PI3K/Akt and Caspase-3 inhibition	Kim et al. [64]
Preclinical in vitro and in vivo	Rat BM-MSCs	Apoptosis in rat INS-1 pancreatic cell line induced by high glucose exposure and in pancreatic β cells in STZ induced type 2 DM in Rat	Not determined	Zhao et al. [54]
Clinical	Human BM-MSCs	Apoptosis and necrosis in alveolar epithelial cells in patients with ARDS	Immunosuppressive and/or trophic factors and/or EVs	Simonson et al. [63]
Preclinical in vitro	Human BM-MSCs	Pyroptosis in human THP-1 monocytic cell line and mouse MH-S alveolar macrophage cell line induced by nanoparticles	IL-10	Naji et al. [48]
Preclinical in vitro	Rat BM-MSCs	Apoptosis and necroptosis in mouse primary cortical neurons induced by deprivation of glucose and oxygen	Caspase-3 and RIP-1/3 inhibition	Kong et al. [57]
Preclinical in vivo	Mouse BM-MSCs	Pyroptosis in mouse hepatocyte induced by d-galactosamine acute liver injury in a mouse model	IL-10	Wang et al. [61]
Preclinical in vitro and in vivo	Rat BM-MSCs	Apoptosis in rat primary cortical neurons induced by deprivation of glucose and oxygen and in rat cortical neurons induced by ischemia with right carotid artery ligation and exposure to hypoxia in a rat model	BDNF, mTOR	Zheng et al. [55]

This table is representative but not exhaustive. Although the table recapitulates studies on MSC death modulation function depending on paracrine factors, this does not exclude the implication of other modes of actions. Studies are ordered from the oldest to the most recent. *BM-MSCs*, bone marrow mesenchymal stem/stromal cells; *UC-MSCs*, umbilical cord mesenchymal stem/stromal cells; *CD*, cluster of differentiation; *IL*, interleukin; *EtOH*, ethanol; *OHDA*, hydroxydopamine; *STZ*, streptozotocin; *RIP*, receptor-interacting protein; *BDNF*, brain-derived neurotrophic factor; *mTOR*, mammalian target of rapamycin

**Table 2:** Mesenchymal stem/stromal cell (MSC) death modulation function depending on gap junctions

Studies	Sources of MSCs	MSC death modulation function	Modes of actions	References
Preclinical in vitro	Mouse BM-MSCs	Apoptosis in rat H9c2 cardiomyoblast cell line induced by deprivation of glucose and oxygen	Cxs and/or TNTs	Cselenyak [41]
Preclinical in vitro and in vivo	Rat BM-MSCs	Apoptosis in rat MSCs induced by deprivation of oxygen and in mouse cardiomyocytes in a myocardial infarction mouse model induced by LAD artery ligation	Cx-43	Wang et al. [67]
Preclinical in vitro and in vivo	Mouse and human BM-MSCs	Apoptosis in human and mouse primary CD34+ cells in vitro, and in vivo in a mouse model of bone marrow transplantation	Cx-43, Cx-45, CXCL12	Schajnovitz et al. [60]
Preclinical in vivo	Mouse and human BM-MSCs	Apoptosis in alveolar epithelial cells induced by LPS in vivo in an acute lung injury mouse model	Cx-43, transfer of mitochondria via EVs and TNTs	Islam et al. [43]
Preclinical in vitro	Human BM-MSCs	Apoptosis in human MM cell lines RPMI 8266, U266, XG-4, XG-7, and human primary MM cells	Cx-43	Zhang et al. [68]
Preclinical in vitro	Human BM-MSCs	Apoptosis and/or necroptosis in human MM cell line RPMI 8266, U266, XG-7, and human primary MM cells induced by bortezomib	Cx-43	Fu et al. [66]

This table is representative but not exhaustive. Although the table recapitulates studies on MSC death modulation function depending on gap junctions, this does not exclude the implication of other modes of actions. Studies are ordered from the oldest to the most recent*. BM-MSCs*, bone marrow mesenchymal stem/stromal cells; *Cxs*, connexins; *CXCL*, CXC ligand; *TNTs*, tunneling nanotubes; *LAD*, left anterior descending; *LPS*, lipopolysaccharide; *EVs*, extracellular vesicles; *MM*, multiple myeloma

**Table 3:** Mesenchymal stem/stromal cell (MSC) death modulation function depending on tunneling nanotubes

Studies	Sources of MSCs	MSC death modulation function	Modes of actions	References
Preclinical in vitro	Human BM-MSCs	Apoptosis in human lung epithelial cell lines A549ρ^0^induced by ethidium bromide	Transfer of mitochondria via EVs and TNTs or both	Spees et al. [27]
Preclinical in vitro	Mouse BM-MSCs	Apoptosis in rat H9c2 cardiomyoblast cell line induced by deprivation of glucose and oxygen	Cxs and/or TNTs	Cselenyak [41]
Preclinical in vivo	Mouse and human BM-MSCs	Apoptosis in alveolar epithelial cells induced by LPS in vivo in an acute lung injury mouse model	Cx-43, transfer of mitochondria via EVs and TNTs	Islam et al. [43]
Preclinical in vitro	Human BM-MSCs	Apoptosis in human primary HUVEC induced by deprivation of oxygen	Transfer of mitochondria via TNTs	Liu et al. [44]
Preclinical in vitro and in vivo	Human BM-MSCs	Apoptosis in human primary bronchial epithelial cells and bronchial smooth muscle cells and human epithelial cell line BEAS-2B, A549 induced by rotenone. Apoptosis in mouse primary tracheal epithelial cells and mouse lung epithelial cell lines ML-12 and lung adenocarcinoma LA-4 induced by rotenone. Apoptosis in alveolar epithelial cells in lung injury mouse models induced by rotenone or an allergen	Transfer of mitochondria via TNTs	Ahmad et al. [38]
Preclinical in vitro	Rat BM-MSCs	Apoptosis in rat cardiomyoblast cell line H9c2 induced by deprivation of glucose and oxygen	Transfer of mitochondria via TNTs	Han et al. [69]
Preclinical in vitro and in vivo	Human ASCs	Apoptosis in human primary cardiomyocytes or endothelial cells induced by ethidium bromide, hydrogen peroxide, or doxorubicin. Apoptosis in mouse cardiomyocytes induced in a myocardial infarction mouse model with LAD artery ligation	Transfer of mitochondria via EVs and/or TNTs, and Heme oxygenase	Mahrouf-Yorgov et al. [46]

This table is representative but not exhaustive. Although the table recapitulates studies on MSC death modulation function depending on tunneling nanotubes, this does not exclude the implication of other modes of actions. Studies are ordered from the oldest to the most recent*. BM-MSCs*, bone marrow mesenchymal stem/stromal cells; *ASCs*, adipose tissue mesenchymal stem/stromal cells; *EVs*, extracellular vesicles; *TNTs*, tunneling nanotubes; *Cxs*, connexins; *LPS*, lipopolysaccharide; *HUVEC*, human umbilical vein endothelial cells; *LAD*, left anterior descending

**Table 4:** Mesenchymal stem/stromal cell (MSC) death modulation function depending on extracellular vesicles

Studies	Sources of MSCs	MSC death modulation function	Modes of actions	References
Preclinical in vitro	Human BM-MSCs	Apoptosis in human A549ρ^0^ lung epithelial cell line induced by ethidium bromide	Transfer of mitochondria via EVs or TNTs or both	Spees et al. [27]
Preclinical in vivo	Mouse and human BM-MSCs	Apoptosis in alveolar epithelial cells induced by LPS in vivo in an acute lung injury mouse model	Cx-43, transfer of mitochondria via EVs and TNTs	Islam et al. [43]
Preclinical in vitro and in vivo	Human BM-MSCs	Apoptosis in human MCF-7 breast cancer cell line and human KHOS osteosarcoma cell line induced by deprivation of serum. Apoptosis in vivo in MCF-7 inoculated in a NU/NU mouse model	Transfer of miRNA-21 and miRNA-34a via EVs	Vallabhaneni et al. [72]
Preclinical in vitro and in vivo	Human BM-MSCs	Apoptosis in human primary MSCs and in mouse RAW 264.7 macrophage cell line induced by oxidative stress and/or silica particles in vitro and in vivo in a mouse silicosis model	Transfer of mitochondria and miRNA-451 via EVs	Phinney et al. [73]
Preclinical in vivo	Human UC-MSCs	Apoptosis in human HFL1 lung fibroblast, HaCAT keratinocyte cell line, and rat primary dermal fibroblasts induced by heat stress in vitro. Apoptosis in rat skin epithelial cells in vivo in a rat burn model	Transfer of Wnt4 via EVs	Zhang et al. [93]
Preclinical in vitro and in vivo	Mouse and human BM-MSCs	Apoptosis in mouse primary bone marrow cells and mouse FDC-P1 hematopoietic cell line induced by γ-irradiation with xenotransplantation in a mouse model	Transfer of miRNA-210-5p, miRNA-106b-3p, and miRNA-155-5p via EVs	Wen et al. [51]
Clinical	Human BM-MSCs	Apoptosis and necrosis in alveolar epithelial cells in patients with ARDS	Immunosuppressive and/or trophic factors and/or EVs	Simonson et al. [63]
Preclinical in vitro and in vivo	Human ASCs	Apoptosis in human primary cardiomyocytes or endothelial cells induced by ethidium bromide, hydrogen peroxide, or doxorubicin. Apoptosis in mouse cardiomyocytes induced in a myocardial infarction mouse model with LAD artery ligation	Transfer of mitochondria via EVs and/or TNTs, and Heme oxygenase	Mahrouf-Yorgov et al. [46]
Preclinical in vitro	Human BM-MSCs	Apoptosis and necroptosis human primary B cell chronic lymphocytic leukemia induced by bortezomib, cladribine, fludarabine, flavopiridol, or methylprednisolone (others)	EVs	Crompot et al. [40]
Preclinical in vitro and in vivo	Human UC-MSCs	Apoptosis in Human L02 hepatocyte cell line induced by hydrogen peroxide or carbon tetrachloride. Apoptosis induced in mouse hepatocyte in vivo by carbon tetrachloride in NU/NU mouse model	Transfer of GPX1 via EVs	Yan et al. [92]
Preclinical in vitro and in vivo	Human iPSC-MSCs	Necroptosis in Human HK-2 epithelial cell line induced by deprivation of oxygen. Necroptosis in renal rat epithelial cells induced in vivo by clamping of renal pedicles in an acute kidney injury rat model	Transfer of SP1 via EVs	Yuan et al. [53]
Preclinical in vitro and in vivo	Mouse BM-MSCs	Apoptosis in mouse primary cardiomyocytes induced by deprivation of oxygen and serum. Apoptosis in mouse cardiomyocytes in vivo in a myocardial infarction mouse model induced by LAD artery ligation	Transfer of miRNA-125b via EVs	Xiao et al. [62]
Preclinical in vitro	Human WJ-MSCS	Apoptosis in mouse Neuro2a neuroblastoma cell line induced by deprivation of glucose and oxygen	Transfer of miRNA let-7a, let-7e, and let-7-5p via EVs	Joerger-Messerli et al. [71]
Preclinical in vitro and in vivo	Human UC-MSCs	Apoptosis in human H1299 and PC-9 lung adenocarcinoma cell lines induced by deprivation of serum. Apoptosis in vivo in human lung H1299 adenocarcinoma cell line in vivo after xenotransplantation in a NU/NU mouse model	Transfer of miRNA-410a via EVs	Dong et al. [70]

This table is representative but not exhaustive. Although the table recapitulates studies on MSC death modulation function depending on extracellular vesicles, this does not exclude the implication of other modes of actions. Studies are ordered from the oldest to the most recent*. BM-MSCs*, bone marrow mesenchymal stem/stromal cells; *ASCs*, adipose tissue mesenchymal stem/stromal cells; *iPSC-MSCs*, inducible pluripotent stem/stromal cell mesenchymal stem cells; *WJ-MSCs*, Wharton’s jelly mesenchymal stem/stromal cells; *UC-MSCs*, umbilical cord mesenchymal stem/stromal cells; *EVs*, extracellular vesicles; *TNTs*, tunneling nanotubes; *Cxs*, connexins; *LPS*, lipopolysaccharide; *miRNA*, microRNA; *LAD*, left anterior descending; *Wnt*, wingless type; *ARDS*, acute respiratory distress syndrome; *GPX*, glutathione peroxidase; *SP*, specificity protein

#### Secreted paracrine factors

**Fig. 1:**
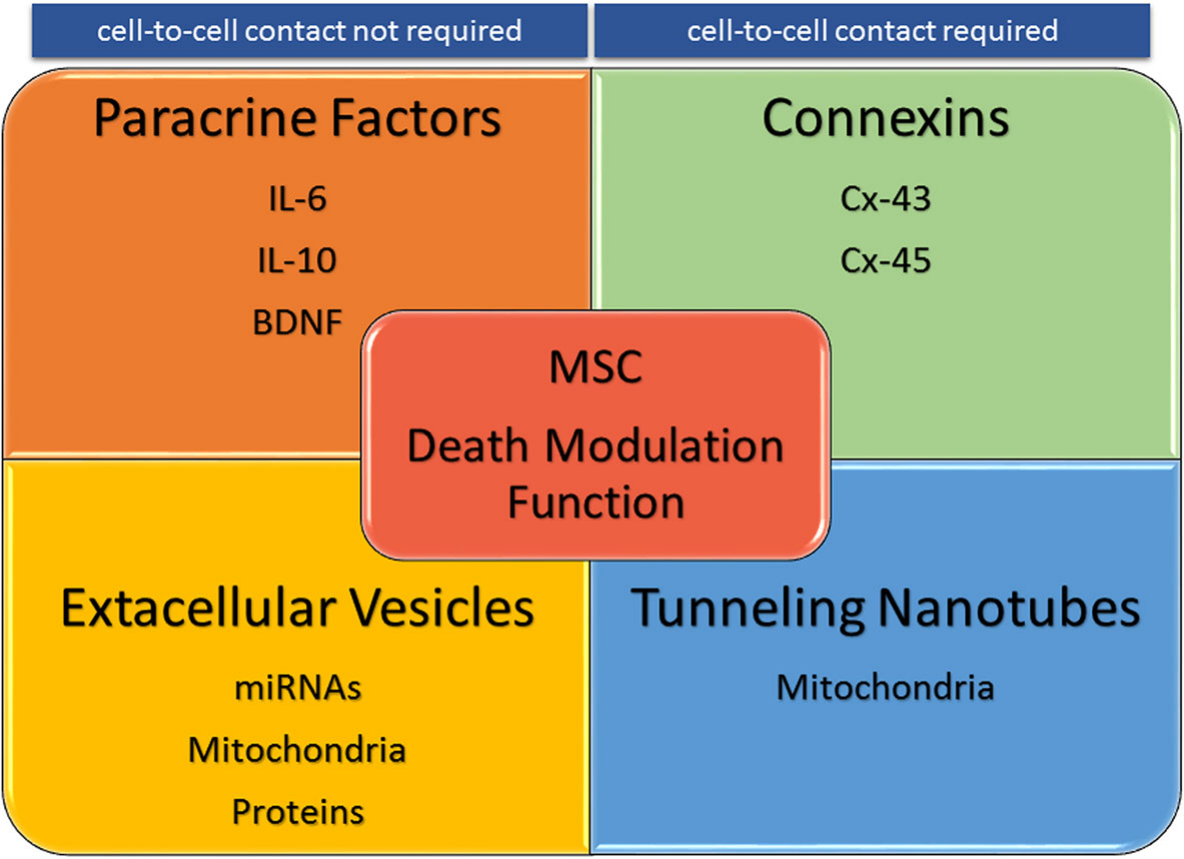
Diagram of possible modes of actions involved in MSC death modulation function. MSC death modulation function might have a mode of action involving paracrine factors (such as IL-6, IL-10, and BDNF) and/or extracellular vesicles (such as for transporting miRNAs, mitochondria, and proteins) were cell-to-cell contact is not required. By contrast, MSC death modulation function might have a mode of action involving connexins (such as Cx-43 and Cx-45) and/or tunneling nanotubes (such as for transporting mitochondria) were cell-to-cell contact is absolutely required. IL, interleukin; BDNF, brain-derived neurotrophic factor; Cx, connexin; miRNAs, microRNAs

There are numerous and solid evidences supporting the role of paracrine factors secreted by MSCs in ameliorating conditions in degenerative and/or inflammatory diseases both in preclinical and clinical settings [23–25, 74–76]. Paracrine factors secreted by MSCs, including chemokines, cytokines, and growth factors, influence the progress of endogenous progenitors’ differentiation such as for angiogenesis or neurogenesis and affect the course of an immune response [25, 74, 76, 77]. All these biological effects may indirectly modulate overall cell death by improving cell survival and/or cell renewal in damaged or diseased tissues [17, 75]. Yet, MSC death modulation function must be considered separately from MSC trophic function and immunosuppression function, as MSC death modulation function would rather have a direct action on death pathways occurring in unfit cells [17]. Hence, paracrine factors secreted by MSCs might participate to MSC death modulation function (Table 1) in different cells subjected to different conditions of RCD. This has been shown mostly in preclinical studies in vitro and in vivo, especially for IL-6 and IL-10 cytokines [48, 58, 61] and brain-derived neurotrophic factor (BDNF) growth factor [55]. Of note, in a preclinical study in vitro, MSCs prevent RCD such as apoptosis of human resting neutrophils and neutrophils stimulated with IL-8 [58]. In this case, MSC death modulation function did not require cell-to-cell contact with unfit neutrophils to rescue cells from apoptosis [58]. The paracrine factor secreted by MSCs that were responsible for neutrophil protection from apoptosis appeared to be IL-6 and involved signaling via the activation of STAT-3 transcription factor [58]. Similarly, IL-10 secreted by MSCs were found to be implicated in the inhibition of RCD, such as pyroptosis in human and mouse macrophages induced by nanoparticles in vitro [48], and of pyroptosis in mouse hepatocytes induced by d-galactosamine in vivo [61]. Furthermore, RCD occurring in rat cortical neurons following hypoxia-ischemia in vitro were prevented when in co-culture with MSCs [55]. As well, in vivo experiments in a rat ischemia model showed that MSC adoptive transfer significantly reduced brain damage [55]. BDNF was identified as a paracrine factor secreted by MSCs responsible for MSC death modulation function in rat cortical neurons suffering ischemia via mammalian target of rapamycin signaling both in vitro and in vivo [55]. Although MSC death modulation function depending on paracrine factors may occur, this does not exclude implication of other modes of actions with either requirement of cell-to-cell contact, such as with Cx gap junctions and/or TNTs, or without requirement of cell-to-cell contact, such as with EVs (Fig. 1).

#### Ca^2+^ ion exchange via Cx gap junctions

Intercellular communications via Cx gap junctions are critically involved in biological processes [78]. Indeed, gap junction channels facilitate the intercellular exchange between cells of ions and small molecules < 1 kDa, influencing cell functions and cell survival in tissues [78]. Hence, preclinical studies showed that BM-MSCs from rat can prevent RCD in H9c2 cardiomyoblasts via direct cell-to-cell interactions, after ischemic damage induced in vitro by oxygen and glucose deprivation [41]. Other preclinical studies of rat BM-MSCs demonstrated specifically that Cx43 gap junctions contribute to BM-MSC survival under hypoxic conditions [67, 79]. Furthermore, upregulation of Cx43 expression by genetic manipulation of those BM-MSCs significantly improved the therapeutic efficacy in a model of myocardial infarction induced by ligation of the left anterior descending (LAD) artery [67, 79]. Concomitantly, BM-MSCs overexpressing Cx43 can produce significantly more pro-survival molecules, such as Bcl-2, and fewer pro-death molecules, such as Bax, which suggests a role for Cx43 in the function of MSCs to prevent RCD processes in cardiomyocytes [67, 79]. Besides, adoptive transfer of mouse or human BM-MSCs into the lung protected against acute lung injury (ALI) induced by lipopolysaccharide in mouse [43]. The mode of action of BM-MSCs that led to improved alveolar cell bioenergetics and reducing cell mortality mostly depended on Cx43 gap junctions. Indeed, the mode of action involved at least Ca^2+^ exchange via Cx43 gap junctions between BM-MSCs and unfit alveolar epithelial cells [43].

Human MSCs can express Cx40, 43, and 45, but the formation of homomeric Cx43 gap junction channels between MSCs themselves and among MSCs and third-party cells is usually detected in vitro in electrophysiological records [80]. Of note, several Cx combinations forming heteromeric gap junction channels may occur, yet these appear to be much less predominant than homomeric Cx43 gap junctions [80]. Thus, homomeric Cx43 channels between human MSCs and cardiomyocytes are functional and might play a role in cell survival [80]. Remarkably, human MSCs can form a dynamic syncytium via Cx43 and Cx45 gap junctions that regulate CXCL12 secretion while favoring survival and homeostasis of HSCs [60]. In a mouse model of BM mononuclear cell transplantation, the mode of action implied Ca^2+^ exchange via Cx43 and Cx45 gap junctions among MSCs that permits signaling by cAMP–protein kinase A and CXCL12 secretion by MSCs, ultimately enhancing the cell survival of HSCs, both in vitro and in vivo [60].

Moreover, in human multiple myeloma (MM) cell lines including RPMI 8266, U266, and XG7, Cx43–gap junctions formed between human MSCs and MM cells reduced bortezomib-induced RCD, such as apoptosis and/or necroptosis, with the function reversible by gap junction inhibitors [66]. Of note, MSCs from MM patients express significantly more Cx43 than do MSCs from healthy individuals, which suggests a role for Cx43 expressed by MSCs in MM cell survival within BM [66, 68]. Hence, RCD occurring in unfit MM cells might be prevented by MSCs via Cx43 gap junction interactions, thereby allowing their growth, but would have rather noxious consequences in MM pathogenesis [66, 68].

#### Transfer of mitochondria via TNTs

Accumulating evidence suggests that MSCs rescue unfit somatic cells by transfer of mitochondria through membrane channels called TNTs [17]. TNTs are biological structures with a diameter of up to 0.7 μm that are sustained by cytoskeleton structures made of f-actin microfilaments and/or α/β-tubulin microtubules [81]. TNTs are thought to support rapid transfer of cellular materials including large organelles from a donor cell to a targeted recipient cell [81]. Intercellular transfer of mitochondria from MSCs to somatic cells was first reported by Spees et al. The authors demonstrated that active mitochondria transfer can rescue aerobic respiration in epithelial A549ρ^0^ cells harboring malfunctioning mitochondria induced by ethidium bromide in vitro [27]. Yet, at that time, the results did not clearly establish whether mitochondria were transferred to unfit epithelial cells solely through TNTs or EVs or both [27]. After adoptive transfer into a rat experimental model of pulmonary diseases induced by cigarette smoke, human MSCs were found to reduce cell death and tissue fibrosis [82]. The reduced lung tissue damage in vivo was associated with transfer of mitochondria from human MSCs to rat airway epithelial cells [82]. As well, transfer of mitochondria from human MSCs to human BEAS-2B bronchial epithelial cells exposed to cigarette smoke in vitro also occurs via TNTs, because inhibition of TNT formation impeded mitochondria transfer, thereby contributing to decreased cell viability [82].

Furthermore, in an ALI mouse experimental model induced by lipopolysaccharide, rat and human MSCs protected against ALI pathology by decreasing RCD occurring in alveolar epithelial cells and by improving alveolar epithelial cell bioenergetics in part via mitochondria transfer through TNTs, both in vitro and in vivo [43]. This finding was further sustained when mitochondria rho GTPase 1 (Miro-1) was identified as a critical factor enabling MSCs to transfer mitochondria through TNT microtubules to unfit tracheal and alveolar epithelial cells, thus decreasing RCD including apoptosis [38]. Indeed, Miro-1 appears essential to facilitate mitochondria trafficking from human MSCs to unfit tracheal and alveolar epithelial cells via TNTs. This process decreased apoptosis in lung epithelial cells in vivo, in mouse models of both rotenone-induced airway injury and allergic airway inflammation. In the same study, the authors underlined that MSC secretion of immunosuppressive factors such as nitric oxide, transforming growth factor β, interleukin 10, and prostaglandin E2 were not significantly involved in the beneficial effects of MSCs in airway injury and inflammation [38]. This last finding suggests and favors a hypothesis considering a less predominant paracrine effect via the MSC immunosuppression function than an MSC death modulation function via TNT mitochondria transfer [38].

Importantly, MSCs donating their own mitochondria via TNTs to unfit somatic cells seem to occur once danger signals from affected cells are sensed and integrated by MSCs, including the sensing of mitochondria released by dying cells [46]. Indeed, human cardiomyoblasts and endothelial cells with RCD induced in vitro by hydrogen peroxide or doxorubicin release their mitochondria that can be engulfed by MSCs, thus initiating MSCs rescuing dying cells [46]. This requirement has been demonstrated in vivo in a mouse model of myocardial infarction induced by LAD artery ligation: inhibition of mitophagy abrogated the MSC death modulation function toward cardiomyocyte apoptosis [46].

#### Transfer of bioactive miRNAs and proteins via EVs

EVs are small membrane vesicles of about 40 nm to 1 μm in diameter that are derived from multivesicular bodies and/or from the plasma membrane of cells. EVs are released by MSCs containing biological materials that can be transferred to targeted recipient cells modifying their biology [83]. This mode of action is thought to carry significant parts of the intercellular communication between MSCs, adult stem cells, progenitors, and somatic cells, both locally and systemically [83]. MSCs produce EVs containing bioactive molecules, such as selective miRNAs, and specific bioactive proteins, such as enzymes [72, 83]. The content of EVs produced by MSCs is likely transferred after EVs fuse with the plasma membrane of targeted recipient cells or after EV endocytosis or phagocytosis by those recipient cells [72, 83]. EVs derived from MSCs have a critical impact on recipient cell biology, particularly improving cell bioenergetics, cell metabolism, and cell survival of unfit adult stem cells and somatic cells [40, 51, 53, 70–73, 84, 85].

The human MSC transfer of miRNAs via EVs ameliorated the silicosis pathophysiology and RCD-mediated lung injury in vivo in a mouse model [73]. Moreover, miRNA transfer via EVs produced by human MSCs alleviated in vivo renal tubular epithelial RCD that can be apoptosis and/or necroptosis induced in vivo by glycerol or cisplatin in severe combined immunodeficient mouse (SCID) [86, 87]. Of note, co-incubation in vitro of EVs derived from human MSCs with human renal tubular epithelial cells injured by cisplatin or by ATP depletion reduced apoptosis by upregulating Bcl-xL, Bcl-2, and BIRC8 pro-survival pathways and downregulating genes involved in RCD, such as caspase-1 and caspase-8 [86, 87]. Mostly, EVs derived from human MSCs likely allow transfer of miRNAs, especially miRNA-24, to renal tubular epithelial cells that modulate cell death pathways ultimately favoring cell survival [86, 88]. Actually, miRNAs transferred by EVs derived from human MSCs to renal tubular epithelial cells were critical in improving conditions in glycerol-induced acute kidney injury in a SCID mouse model; indeed, depletion of miRNAs using a knockdown method (Drosha knockdown) abolished the therapeutic efficacy of EVs [88]. Furthermore, EVs derived from human MSCs containing miRNA-22 and its transfer to unfit mouse neonatal cardiomyocytes abrogated RCD by apoptosis induced by ischemic stress via direct interactions with methyl CpG binding protein 2 [89]. As well, EVs derived from human MSCs and containing miRNA-22 significantly reduced fibrosis in a myocardial infarction mouse model induced by LAD artery ligation [89].

Likewise, human MSC-derived EVs contain miRNA let-7 precursors that can mature to let-7a, let-7e, and let-7-5p miRNAs. These let-7a, let-7e, and let-7-5p miRNAs upregulated Bcl-2 and downregulated caspase-3 and prevented RCD such as apoptosis and autophagy-related cell death in MSCs themselves and in the mouse neuron cell line Neuro2a in vitro when subjected to RCD cell signaling after deprivation of oxygen and glucose [71]. In addition, inhibition of miRNA-21 and miRNA-34a produced by human MSCs deprived of serum increased the apoptosis of MSCs, which suggests an essential role of miRNA-21 and miRNA-34a in preventing RCD in MSCs [72]. Besides, EVs derived from MSCs containing miRNA-21 and miRNA-34a inhibited apoptosis in MCF-7 breast cancer cells and KHOS osteosarcoma cells subjected to RCD signaling both in vitro and in vivo [72]. Similarly, EVs containing miRNA-410 derived from MSCs promoted the growth of lung adenocarcinoma cancer cells in vivo that was associated with downregulation of phosphatase and tensin homolog protein expression, with a decrease in RCD such as cell apoptosis [70].

Of note, the authors of a preclinical study demonstrated that EVs derived from MSCs transported neprilysin, an amyloid β (Aβ)-degrading enzyme, capable of reducing both secreted and intracellular Aβ molecules in mouse Neuro2a cells in vitro [90]. Hence, EV delivery of neprilysin to affected neurons with accumulation of Aβ peptides could potentially protect against Aβ-induced caspase-1-dependent neuron pyroptosis involved in Alzheimer’s disease [90]. Moreover, EVs derived from human MSCs alleviated liver fibrosis and hepatocyte RCD in vivo in a mouse model of acute liver injury induced by carbon tetrachloride (CCl_4_) peritoneal injection [91]. Especially, EVs derived from human MSCs carrying glutathione peroxidase 1 (GPX1) prevented hepatocyte apoptosis and/or necroptosis in a mouse with liver injury induced by CCl_4_ [92]. GPX1 knockdown revoked the anti-RCD abilities of EVs derived from human MSCs both in vitro and in vivo [92]. Furthermore, EVs derived from human MSCs ameliorated cutaneous healing of skin burn in a rat model [93]. This observation was associated with inhibited RCD by apoptosis in rat epithelial cells via transfer of biologically active Wnt4 to injured cells both in vitro and in vivo [93]. As well, human keratinocytes, HaCATs, after in vitro heat stress-induced RCD, were rescued from apoptosis by EVs derived from human MSCs via transfer of Wnt4 into unfit HaCATs [93]. Similarly, with human-induced pluripotent-derived mesenchymal stem/stromal cells (iPSC-MSCs), it has been shown that EVs produced by iPSC-MSCs contained specificity protein 1 (SP1) transcription factor. iPSC-MSC EV transfer of SP1 to human HK-2 epithelial cells undergoing necroptosis induced by deprivation of oxygen rescues the HK-2 through transcriptional activating of sphingosine kinase 1 [53]. As well, transfer of SP1 via EVs appears critical to inhibit necroptosis occurring in rat renal epithelial cells in vivo in a rat model of acute kidney injury induced by renal pedicle clamping [53].

In addition to MSCs transferring miRNAs and/or proteins via EVs to unfit somatic cells, MSCs may also transfer larger organelles such as mitochondria via EVs to modulate RCD in unfit cells. Indeed, this pathway has been suggested to play a critical role in the transfer of mitochondria to injured alveolar epithelial cells [43, 63]. The transfer would allow for amelioration of the pathogenesis of acute respiratory distress syndrome in vivo in both preclinical and clinical settings [63, 94]. The MSC death modulation function may be exploited in the clinic for degenerative and/or inflammatory diseases. For example, in a clinical assay, bronchoalveolar lavage fluid of two patients with acute respiratory distress syndrome who received adoptive transfer of MSCs showed a rapid decrease in levels of cell death biomarkers, including biomarkers of apoptosis and necrosis of alveolar epithelial cells [63]. Fluids were assessed within just a few hours after adoptive transfer of MSCs in both patients and notably several hours before a decrease in inflammatory biomarkers were detected [63]. This finding might indicate that the MSC death modulation function is implemented early and therefore is crucial in the beneficial effect observed in this clinical assay [17, 63].

### Are gap junctions, TNTs, and EVs synergistic to convey MSC death modulation function?

**Fig. 2:**
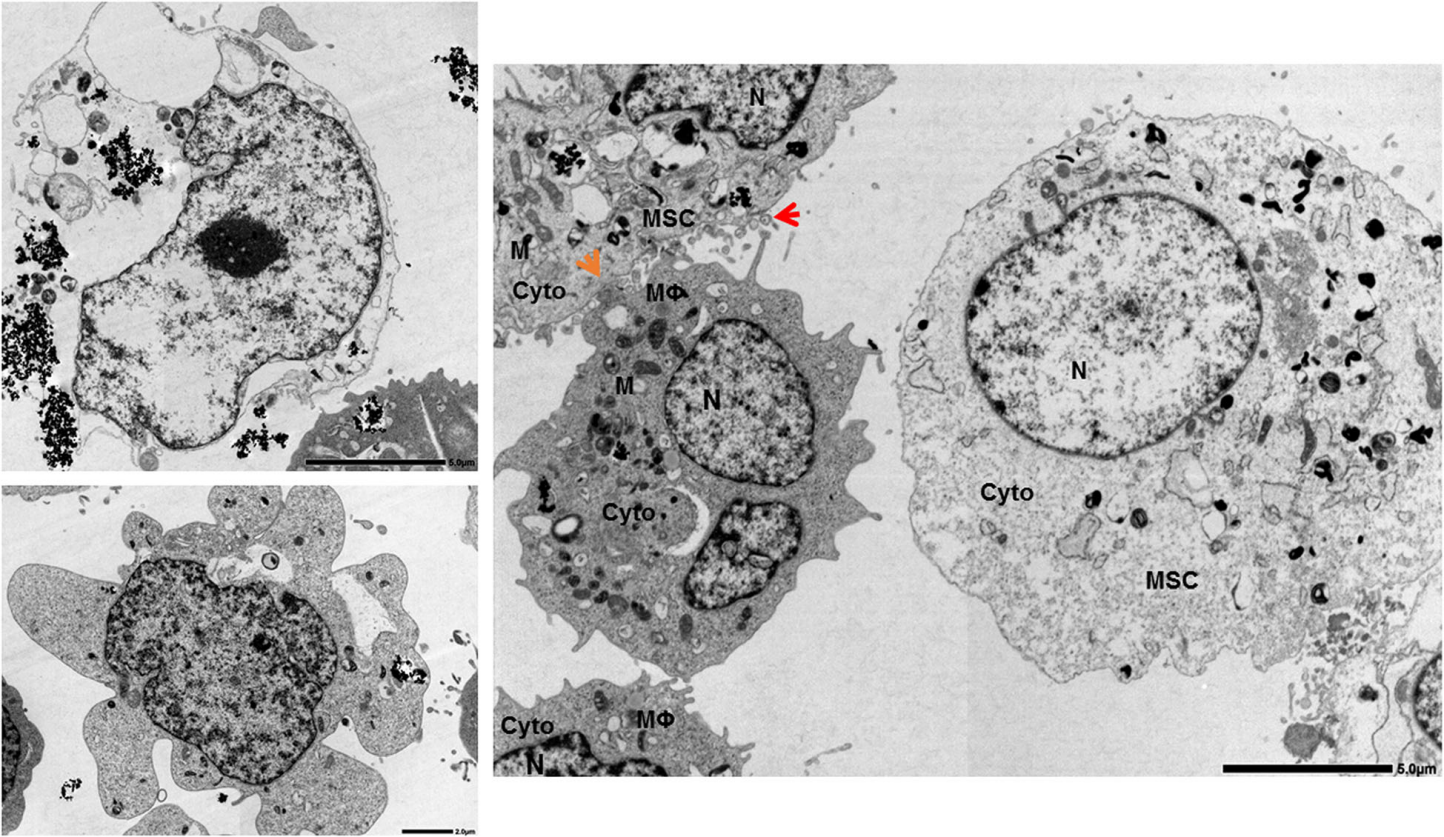
Transmission electron micrography of regulated cell death (RCD) in human macrophages and cell-to-cell interactions with human bone marrow mesenchymal stem cells. A human macrophage exposed to metallic nanoparticles undergoing (top left) pyroptosis, with an intact nucleus and disrupted plasma membrane, and (bottom left) apoptosis, featuring an intact plasma membrane and formation of membrane blebs. Right, co-culture of macrophages and MSCs in the presence of metallic nanoparticles. Shows human monocyte-derived macrophages (phorbol-12-myristate-13-acetate–activated THP-1 cells) in close contact with mesenchymal stem/stromal cells (MSCs). MSCs appear as large cells with clear cytoplasm, morphologically distinguishable from macrophages. The orange arrow indicates a tight membrane contact between an MSC and a macrophage, and the red arrow the presence of extracellular vesicles (~ 100–200 nm in size) near the cell-to-cell contact. Mitochondria in both cells appear co-localized to the side of the cell-to-cell contact. Macrophage to MSC ratio is 2:1. Left, original magnification × 3000. Right, × 2000 of macrophage to MSC co-culture 12 h after pyroptosis induction in macrophages; scale bars are 5 μm (top left), 2 μm (bottom left), 5 μm (right). One original representative image in 3 is shown (Naji Lab). Cyto, cytoplasm; N, nucleus; M, mitochondria; MΦ, macrophage; MSC, mesenchymal stem/stromal cell

The MSC death modulation function appears to contribute a part of the therapeutic effects of MSCs for various diseases [17]. This function is implemented predominantly by MSCs through modes of actions implying direct cell-to-cell interactions requiring either cell contact with gap junctions and TNTs, or no cell contact with EVs (Fig. 1). Together, these modes of actions might be interdependent in ultimately controlling the transfer of mitochondria from MSCs to unfit cells for the full potency of the MSC death modulation function. Indeed, recent studies suggested that Cx43 gap junctions contribute to facilitate TNT- and EV-dependent exchange of biological materials between cells [78]. Thus, the formation of Cx43 gap junctions between MSCs and unfit cells may serve as a beacon by first sensing cell needs via Ca^2+^ and by directing the transfer of larger biological materials such as proteins and organelles through TNTs and EVs in a circumscribed microenvironment between cells. Therefore, the three individual modes of actions described above likely function synergistically in conveying the MSC death modulation function (Fig. 2).

## Conclusion

MSCs could be a remarkable adult stem cell source for cell therapy and advantageous for responding to various medical demands. Yet, there are obstacles to the development of MSC therapy [8, 9, 15, 17, 26, 76, 95–97]. In some clinical trials of MSC therapy, the clinical benefits were mitigated [15, 76, 95, 96], but in others, the outcome of MSC therapy was more encouraging [63, 98–104]. Thus, MSC therapy must be consolidated by refining its efficacy and consistency in therapy of human disorders. A better understanding of MSC biological functions could help assess the potency of MSCs in therapeutics and could unlock the knowledge of MSC identity. This understanding includes the MSC death modulation function in particular, which is just being revealed, and whose potential relevance in degenerative and/or inflammatory diseases could be critical. The expression of Cx-43 as well as the mitochondria bioenergetic (Δψm) and/or mitochondria phenotype (Miro-1) might be good indicators of functional potency for the MSC death modulation function of ex vivo-cultured MSCs and useful biomarkers for identifying MSCs for clinical use.

## Abbreviations

AKI: Acute kidney injury
ALI: Acute lung injury
ARDS: Acute respiratory distress syndrome
ASCs: Adipose tissue mesenchymal stem/stromal cells
BDNF: Brain-derived neurotrophic factor
BM-MSCs: Bone marrow mesenchymal stem/stromal cells
CD: Cluster of differentiation
CXCL: CXC ligand
Cxs: Connexins
EtOH: Ethanol
EVs: Extracellular vesicles
GPX: Glutathione peroxidase
HUVEC: Human umbilical vein endothelial cells
IL: Interleukin
iPSC-MSCs: Inducible pluripotent stem/stromal cell–mesenchymal stem cells
LAD: Left anterior descending
LPS: Lipopolysaccharide
miRNA: MicroRNA
MM: Multiple myeloma
mTOR: Mammalian target of rapamycin
OHDA: Hydroxydopamine
RIP: Receptor-interacting protein
SP: Specificity protein
STZ: Streptozotocin
TNTs: Tunneling nanotubes
UC-MSCs: Umbilical cord mesenchymal stem/stromal cells
WJ-MSCs: Wharton’s jelly mesenchymal stem/stromal cells
Wnt: Wingless type
